# Characterization of Cancer Stem Cells in Moderately Differentiated Buccal Mucosal Squamous Cell Carcinoma

**DOI:** 10.3389/fsurg.2016.00046

**Published:** 2016-08-02

**Authors:** Helen H. Yu, Therese Featherston, Swee T. Tan, Alice M. Chibnall, Helen D. Brasch, Paul F. Davis, Tinte Itinteang

**Affiliations:** ^1^Gillies McIndoe Research Institute, Wellington, New Zealand; ^2^Wellington Regional Plastic, Maxillofacial and Burns Unit, Hutt Hospital, Wellington, New Zealand

**Keywords:** buccal, mucosal, squamous cell carcinoma, cancer, stem cells, oral cavity

## Abstract

**Aim:**

To identify and characterize cancer stem cells (CSC) in moderately differentiated buccal mucosa squamous cell carcinoma (MDBMSCC).

**Methods:**

Four micrometer-thick, formalin-fixed, paraffin-embedded MDBMSCC samples from six patients underwent 3,3-diaminobenzidine (DAB) immunohistochemical (IHC) staining for the embryonic stem cell (ESC) markers, NANOG, OCT4, SALL4, SOX2, and pSTAT3; cancer stem cell marker, CD44; squamous cell carcinoma (SCC) marker, EMA; and endothelial marker, CD34. The transcriptional activities of the genes encoding NANOG, OCT4, SOX2, SALL4, STAT3, and CD44 were studied using NanoString gene expression analysis and colorimetric *in situ* hybridization (CISH) for NANOG, OCT4, SOX2, SALL4, and STAT3.

**Results:**

Diaminobenzidine and immunofluorescent (IF) IHC staining demonstrated the presence of (1) an EMA^+^/CD44^+^/SOX2^+^/SALL4^+^/OCT4^+^/pSTAT3^+^/NANOG^+^ CSC subpopulation within the tumor nests; (2) an EMA^−^/CD44^−^/CD34^−^/SOX2^+^/OCT4^+^/pSTAT3^+^/NANOG^+^ subpopulation within the stroma between the tumor nests; and (3) an EMA^−^/CD44^−^/CD34^+^/SOX2^+^/SALL4^+^/OCT4^+^/pSTAT3^+^/NANOG^+^ subpopulation on the endothelium of the microvessels within the stroma. The expression of CD44, SOX2, SALL4, OCT4, pSTAT3, and NANOG was confirmed by the presence of mRNA transcripts, using NanoString analysis and NANOG, OCT4, SOX2, SALL4, and STAT3 by CISH staining.

**Conclusion:**

This study demonstrated a novel finding of three separate CSC subpopulations within MDBMSCC: (1) within the tumor nests expressing EMA, CD44, SOX2, SALL4, OCT4, pSTAT3, and NANOG; (2) within the stroma expressing SOX2, SALL4, OCT4, pSTAT3, and NANOG; and (3) on the endothelium of the microvessels within the stroma expressing CD34, SOX2, SALL4, OCT4, pSTAT3, and NANOG.

## Introduction

Oral cavity cancer is the sixth most common cancer worldwide (1), with over 90% being squamous cell carcinoma (SCC) (1). Oral cavity squamous cell carcinoma (OCSCC) may arise from sites of leukoplakia or erythroplakia involving the oral squamous epithelium (2, 3), characterized by its dysplastic morphology. Late-stage disease often presents with nodal metastases and less commonly, metastasis to the lung, brain, bone, and liver (2, 3).

Oral cavity squamous cell carcinoma affects the oral tongue, floor of the mouth, buccal mucosa, hard palate, mandibular and maxillary alveolus, vestibule of mouth, and retromolar trigone (4, 5). While buccal mucosal squamous cell carcinoma (BMSCC) accounts for <10% of all OCSCC in the Western world (4, 6), it is the most prevalent OCSCC in South East Asia and South Asia (6, 7). This high prevalence is primarily due to the practice of betel nut chewing (6, 7), and/or alcohol consumption (8) and/or tobacco use (5), although recent studies suggest other non-lifestyle contributing factors (5). Due to the particularly aggressive nature of this cancer (9), patients with BMSCC have a high locoregional recurrence rate of up to 57% with associated poor survival (10). Despite improvements in diagnosis and treatments, 5-year survival rates have remained around 50% since the early 1980s (4–7). Current treatment of BMSCC involves a multimodal approach (11), usually requiring surgical resection with postoperative adjuvant radiotherapy (RT) and occasionally chemotherapy (ChT), especially in cases with adverse features, such as positive surgical margins and/or extracapsular spread of the nodal metastases (11).

It has been proposed that the development and spread of many cancers (12–14), including OCSCC (15), are driven by a subpopulation of cancer cells known as cancer stem cells (CSC) (12). Recent studies have identified these CSC in OCSCC by their expression of the embryonic stem cell (ESC) markers, NANOG (15), SOX2 (16), SALL4 (17), phosphorylated (activated) form of signal transducer and activator of transcription 3 (pSTAT3) (18), and OCT4 (15, 16). NANOG is a transcription factor that controls cell proliferation, migration, and invasion (19). SOX2 is critical for the self-renewal properties of ESC (16). OCT4 is a POU domain transcription factor associated with cell self-renewal, proliferation, and pluripotency (16) and works synergistically with SOX2 to regulate pluripotency of ESC (19). This pluripotency network also includes additional factors, such as SALL4, a transcriptional activator of POU5f1 that maintains embryonic pluripotency by modulating the expression of OCT4 (20), and pSTAT3, which is required for tumor formation and growth, and suppression of apoptosis (21). The cell surface marker CD44 is a multistructural and multifunctional CSC marker associated with angiogenesis, cell proliferation, migration, and differentiation (22), as well as some progenitor cell properties (23). Unlike progenitor cells and differentiated cells, CSCs possess the ability for self-renewal and multilineage differentiation through either asymmetric or symmetric cell division (12). It has been reported that the presence of CSC is associated with both a greater capacity for tumor growth and a worsening prognosis (13, 15). This is supported by the idea that CSC have unique abilities to resist cell damage and may represent critical mediators for both RT and ChT resistance (24).

Despite recent literature showing the presence of CSC within many cancers, including breast (25), brain (26), and pancreatic (27) cancer, there have been no reports describing the presence of CSC within BMSCC.

This study investigated the expression, within moderately differentiated buccal mucosa squamous cell carcinoma (MDBMSCC), of the ESC markers NANOG, SOX2, SALL4, pSTAT3, OCT4, and CD44, at both the transcriptional and translational level, to identify and characterize the putative CSC population.

## Materials and Methods

### Tissue Samples

Previously untreated MDBMSCCs from five male and one female patients, aged 38–80 (mean, 59) years, were used in this study, which was approved by the Central Health and Disabilities Ethics Committee (ref. no. 12/CEN/74).

### Histochemical Staining and Immunohistochemical Staining

Hematoxylin and eosin staining was performed on 4 μm-thick, formalin-fixed, paraffin-embedded blocks of six MDBMSCC samples, to confirm the appropriate histological grading and the presence of BMSCC in the sections. 3,3-Diaminobenzidine (DAB) immunohistochemical (IHC) staining of the sections was then performed using the Leica Bond Rx auto-stainer (Leica, Nussloch, Germany), as previously described (28). Staining for NANOG (1:2000; cat# NBP1-04320, Novus Biologicals LLC, Littleton, CO, USA), SOX2 (1:500; cat# PA-094, Thermo Fisher Scientific, Rockford, IL, USA), SALL4 (1:30; cat# CM385M-16, Cell Marque, Rocklin, CA, USA), pSTAT3 (1:100; cat# 9145, Cell Signaling Technology, Danvers, MA, USA), and OCT4 (1:200; cat# NBP1-47923, Novus Biologicals LLC, Littleton, CO, USA), CD34 (ready-to-use; cat# PA0212, Leica), CD44 (1:1500; cat# MRQ-13, Cell Marque), and epithelial membrane antigen (EMA, ready-to-use; cat# PA0212, Leica), diluted with Bond™ primary antibody diluent (Leica AR9352), was done for all tissue samples.

To confirm co-expression of two proteins, representative slides of MDBMSCC (*n* = 2) underwent immunofluorescent (IF) IHC staining using a combination of Vectafluor Excel anti-rabbit 594 (ready-to-use; cat# VEDK-1594, Vector Laboratories, Burlingame, CA, USA) and Alexa Fluor anti-mouse 488 (1:500; cat# A21202, Life Technologies) so as to detect combinations that included NANOG, SOX2, and pSTAT3, and Vectafluor Excel anti-mouse (ready-to-use; cat# VEDK2488, Vector Laboratories) and Alexa Fluor anti-rabbit 594 (1:500; cat# A21207, Life Technologies) to detect combinations that included CD44, EMA, OCT4, or SALL4.

Positive control tissues used for the primary antibodies were human infantile hemangioma (IH) for NANOG (29), and SALL4 (30), myometrium for OCT4 (31), skin for SOX2 (32), tonsil for pSTAT3 (33) and CD44 (34). A negative control for the primary antibody was performed on a MDBMSCC sample from the cohort of patients used for IHC staining.

### Nanostring Gene Expression Analysis

RNA was extracted from five snap-frozen MDBMSCC samples from the same cohort of patients used for DAB IHC staining and was used for NanoString nCounter™ Gene Expression Assay (Nanostring Technologies, Seattle, WA, USA). Total RNA was extracted using the MagJET RNA kit (Thermo Fisher Scientific) with the protocol adapted for tissue and run on a KingFisher Duo machine (Thermo Fisher Scientific). RNA samples were then quantitated on a Qubit^®^ 2.0 fluorometer (Invitrogen, Life Technologies) and were subject to RNA integrity analysis *via* the 2100 Bioanalyzer Instrument (Agilent Technologies). The samples then underwent NanoString nCounter gene expression assay performed by New Zealand Genomics (Dunedin, New Zealand) according to the manufacturer’s protocol. Probes for the genes encoding NANOG (NM_024865.2), SALL4 (NM_020436.3) SOX2 (NM_003106.2), OCT4 (NM_002701.4), CD44 (NM_001001392.1), and STAT3 (NM_139276.2) and the housekeeping gene GUSB (NM_000181.1) were designed and manufactured by NanoString Technologies. Raw data were analyzed using nSolver™ software (NanoString Technologies) using standard settings and normalized against the housekeeping gene.

### Colorimetric *In Situ* Hybridization

Representative 4 μm-thick, formalin-fixed, paraffin-embedded sections of three MDBMSCC samples from the original cohort of six patients used for DAB IHC staining were used for mRNA colorimetric *in situ* hybridization (CISH). Staining was done on the Leica Bond Rx auto-stainer and detected using the ViewRNA red stain kit (Affymetrix, Santa Clara, CA, USA), as previously described (35). The probes used for NANOG (NM_024865), SOX2 (NM_003106), SALL4 (NM_020436), STAT3 (NM_003150), and OCT4 (NM_002701) were obtained from Affymetrix. Positive controls used were human IH for SALL4 and human seminoma for NANOG, SOX2, OCT4, and STAT3. A negative control for the primary antibody was done on a sample of MDBMSCC from the CISH cohort by omitting the probe.

### Image Analysis

Diaminobenzidine IHC- and CISH-stained slides were viewed and imaged using an Olympus BX53 light microscope (Tokyo, Japan). IF IHC-stained images were captured using an Olympus FV1200 biological confocal laser-scanning microscope and processed with the cellSens Dimension 1.11 software using 2D deconvolution algorithm (Olympus).

## Results

### Histochemical and Immunohistochemical Staining

Hematoxylin and eosin staining confirmed the appropriate histological grading and the presence of MDBMSCC on the slides used for DAB IHC staining (data not shown).

**Figure 1:**
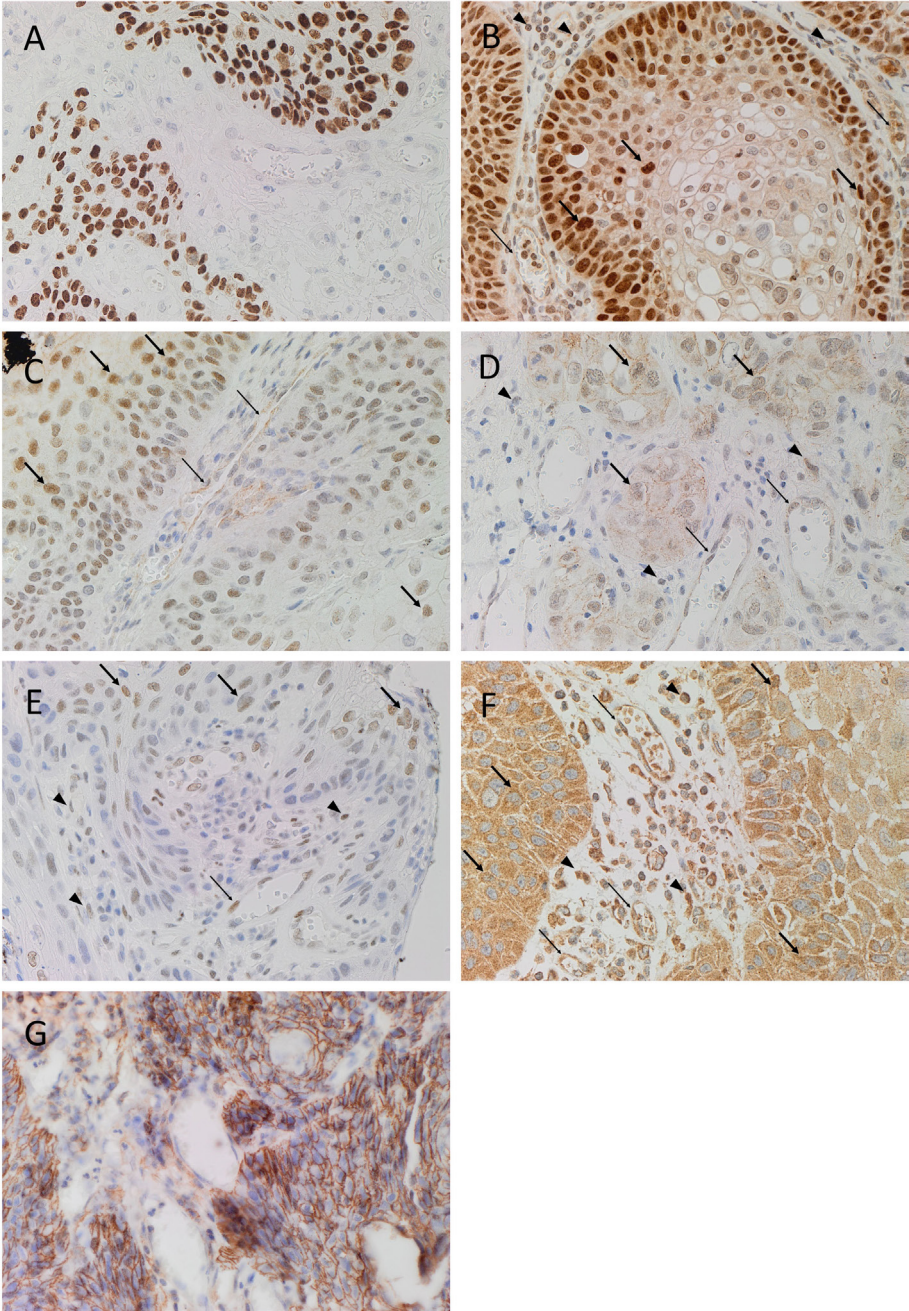
**Representative DAB IHC-stained sections of MDBMSCC demonstrating nuclear expression of EMA of cells within the tumor nests [(A), brown]**. Expression of SOX2 was seen in cells within the tumor nests [**(B)**, brown, *thick arrows*] and the stroma [**(B)**, brown, *arrowheads*], and on the endothelium of the microvessels within the stroma [**(B)**, brown, *thin arrows*]. Expression of SALL4 was limited to cells within the tumor nests [**(C)**, brown, *thick arrows*] and the endothelium of the microvessels [**(C)**, brown, *thin arrows*]. OCT4 was also expressed in cells within tumor nests [**(D)**, brown, *thick arrows*] and the stroma [**(D)**, brown, *arrowheads*], and the endothelium of the microvessels within the stroma [**(D)**, brown, *thin arrows*]. Expression of pSTAT3 was detected on cells within the tumor nests [**(E)**, brown, *thick arrows*] and the stroma [**(E)**, brown, *arrowheads*], and the endothelium of the microvessels within the stroma [**(E)**, brown, *thin arrows*]. NANOG was also seen in cells within the tumor nests [**(F)**, brown, *thick arrows*] and the stroma [**(F)**, brown, *arrowheads*], and the endothelium of the microvessels within the stroma [**(F)**, brown, *thin arrows*]. CD44 expression was seen as membranous staining of the tumor nest cells [**(G)**, brown]. Original magnification: 400×.

Diaminobenzidine IHC staining showed that cells within the tumor nests stained positively with the SCC marker EMA (Figure 1A, brown). Both SOX2 (Figure 1B, brown) and SALL4 (Figure 1C, brown) showed positive nuclear staining with increased expression especially toward the periphery of the tumor nests. Nuclear staining for OCT4 (Figure 1D, brown) and pSTAT3 (Figure 1E, brown) of the cells within the tumor nests was noted, while primarily cytoplasmic staining with some nuclear positivity for NANOG (Figure 1F, brown) was observed. There was also membranous expression of the CSC marker CD44 (Figure 1G, brown) that was exclusively localized to cells within the tumor nests. Clusters of cells within the stroma also showed nuclear expression of SOX2 (Figure 1B, brown, *arrowheads*), OCT4 (Figure 1D, brown, *arrowheads*), and pSTAT3 (Figure 1E, brown, *arrowheads*), while cytoplasmic and nuclear expression of NANOG (Figure 1F, brown, *arrowheads*) was observed in these cells. The endothelium of the microvessels within the stroma also stained positively for SOX2 (Figure 1B, brown, *thin arrows*), SALL4 (Figure 1C, brown, *thin arrows*), OCT4 (Figure 1D, brown, *thin arrows*), pSTAT3 (Figure 1E, brown, *thin arrows*), and NANOG (Figure 1F, brown, *thin arrows*).

Expected staining patterns for the NANOG (Figure S1A in Supplementary Material, brown), SALL4 (Figure S1B in Supplementary Material, brown), OCT4 (Figure S1C in Supplementary Material, brown), SOX2 (Figure S1D in Supplementary Material, brown), pSTAT3 (Figure S1E in Supplementary Material, brown), and CD44 (Figure S1F in Supplementary Material, brown) were demonstrated in the respective positive controls. The omission of the primary antibody in MDBMSCC samples provided an appropriate negative control (Figure S1G in Supplementary Material).

**Figure 2:**
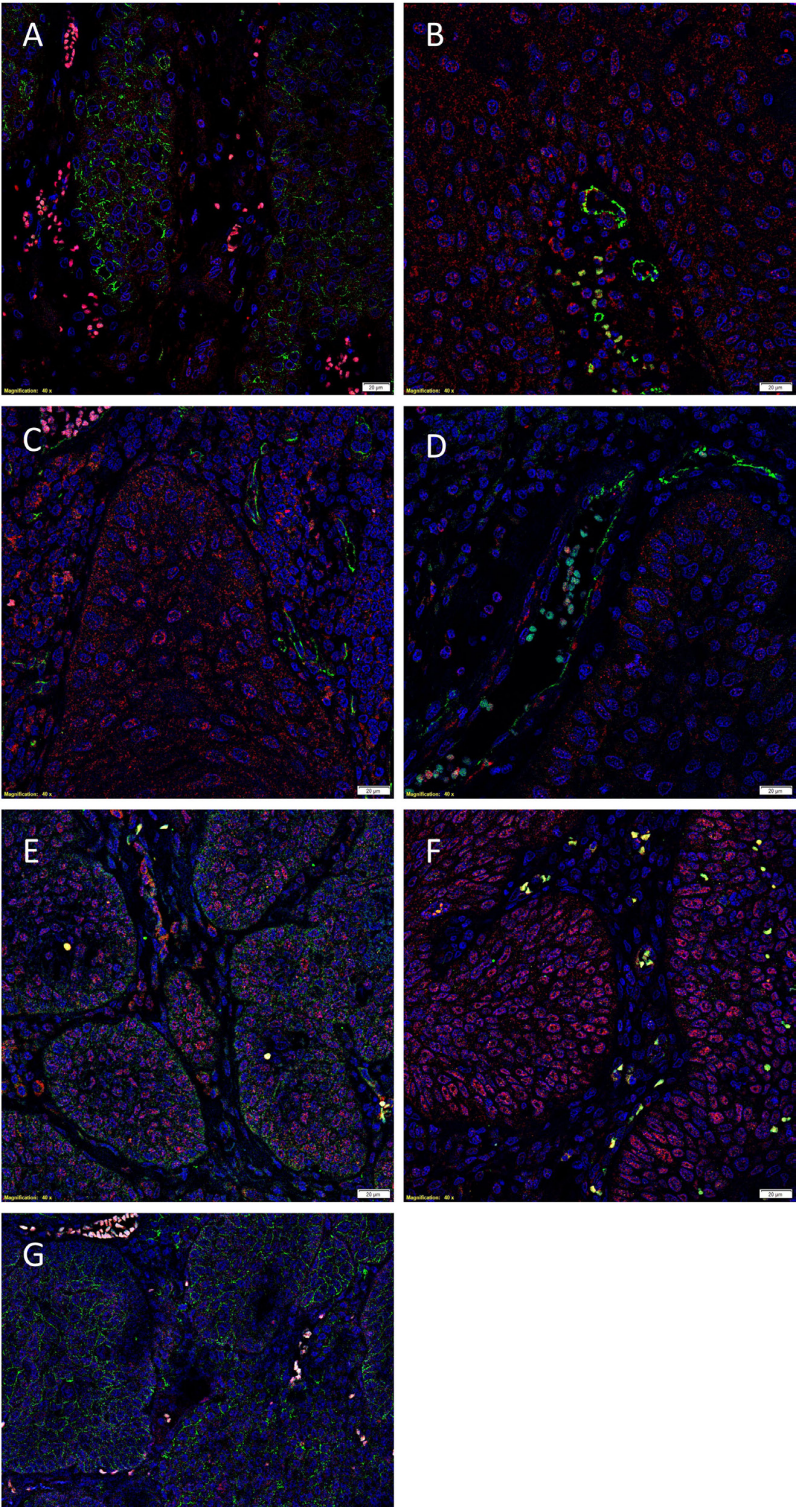
**Representative IF IHC-stained sections of MDBMSCC demonstrating the expression of pSTAT3 [(A), red] and EMA [(A), green] by cells within the tumor nests**. There was a CSC subpopulation remonstrating nuclear co-expression of STAT3 [**(B)**, red] and CD34 [**(B)**, green], appearing as orange, on the endothelium of the microvessels within the stroma; and another subpopulation staining only positively for pSTAT3 within the stroma [**(B)**, red]. Nuclear expression of NANOG [**(C)**, red] was demonstrated on the endothelium of the microvessels which expressed CD34 [**(C)**, green] within stroma. The NANOG^+^ cells [**(C)**, red] that do not express CD34 were seen within the tumor nests and the stroma. SOX2 [**(D)**, red] was also expressed by cells within tumor nests and the stroma, and the endothelium of the microvessels expressing CD34 [**(D)**, green]. Nuclear expression of both SOX2 [**(E)**, red] and SALL4 [**(E)**, green], appearing as orange, was seen on the cells within the tumor nests and the stroma. Expression of both SOX2 [**(F)**, red] and OCT4 [**(F)**, green], appearing as orange, was seen on cells within the tumor nests and the stroma, and the endothelium of the microvessels within the stroma. pSTAT3 [**(G)**, red] and membranous staining CD44 [**(G)**, green] were co-expressed by cells within the tumor nests. Scale bars: 20 μm.

To demonstrate co-expression of the ESC markers, IF IHC staining was performed on two representative MDBMSCC samples used for DAB IHC staining. pSTAT3 staining (Figures 2A,B, red) was localized to cells within the tumor nests that displayed membranous staining for EMA (Figure 2A, green), the endothelium of the microvessels that stained positively for CD34 (Figure 2B, green) and cells within the stroma. NANOG was expressed by cells within the tumor nests (Figure 2C, red) and the stroma (Figure 2C, red), as well as the endothelium of the microvessels that stained positively for CD34 (Figure 2C, green). A similar staining pattern was also seen with SOX2 for cells within the tumor nests (Figure 2D, red) and the stroma (Figure 2D, red), and the endothelium marked by CD34 (Figure 2D, green). SOX2 (Figure 2E, red) and SALL4 (Figure 2E, green) were co-expressed (appearing as orange) by cells within the tumor nests and the stroma (Figure 2E), and the endothelium of the microvessels (Figure 2E, red). SOX2 (Figure 2F, red) and OCT4 (Figure 2F, green) were also co-localized (appearing as orange) to cells within the tumor nests and the stroma (Figure 2F), and the endothelium of the microvessels (Figure 2F). pSTAT3 (Figure 2G, red) and membranous staining of CD44 (Figure 2G, green) were co-expressed by cells within the tumor nests. Images of the individual stains are presented in Figure S2 in Supplementary Material.

### NanoString Gene Analysis

**Figure 3:**
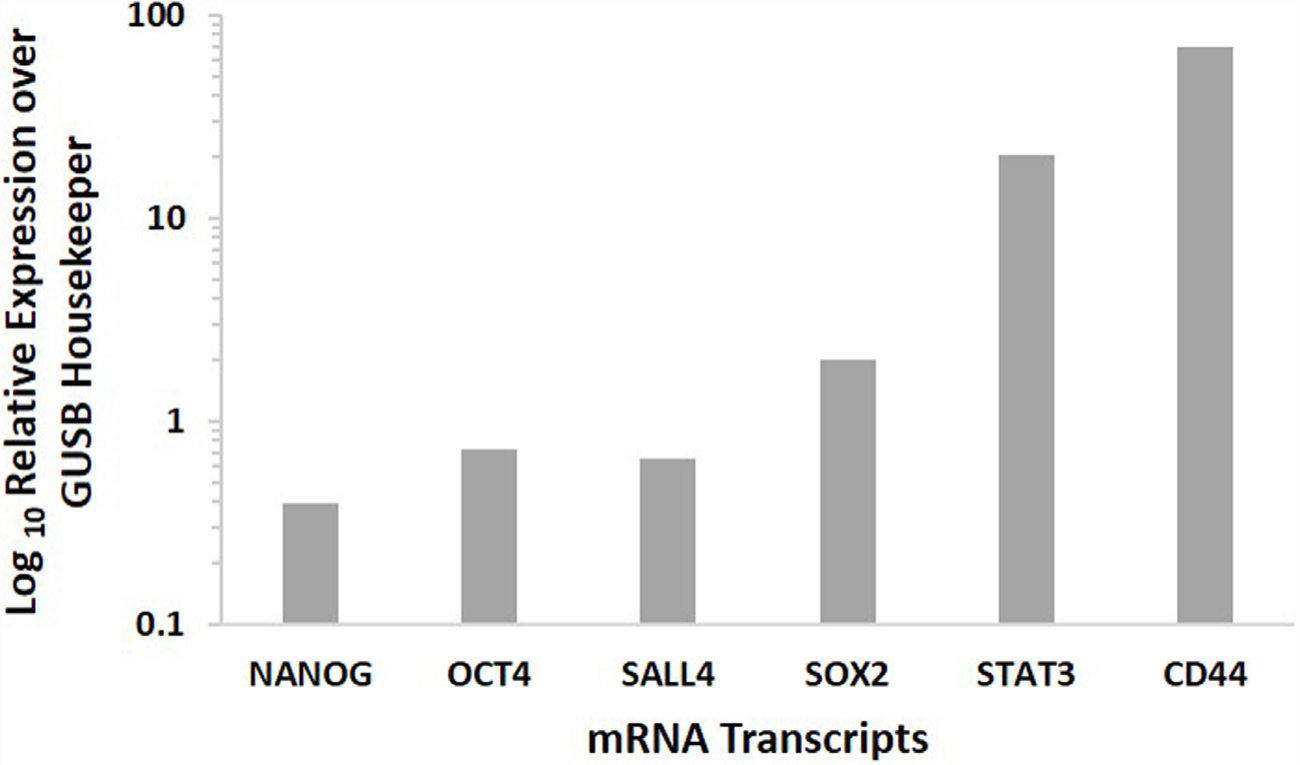
**Expression of CSC-related mRNA transcripts of NANOG, OCT4, SALL4, SOX2 STAT3, and CD44 in MDBMSCC samples from five patients**. Their expression was normalized over GUSB housekeeper.

NanoString analysis confirmed the presence of the mRNA transcripts for NANOG, OCT4, SALL4, SOX2, STAT3, and CD44, in MDBMSCC samples of all five patients, while SALL4 was detected in four of the five samples (Figure 3).

### Colorimetric *In Situ* Hybridization

**Figure 4:**
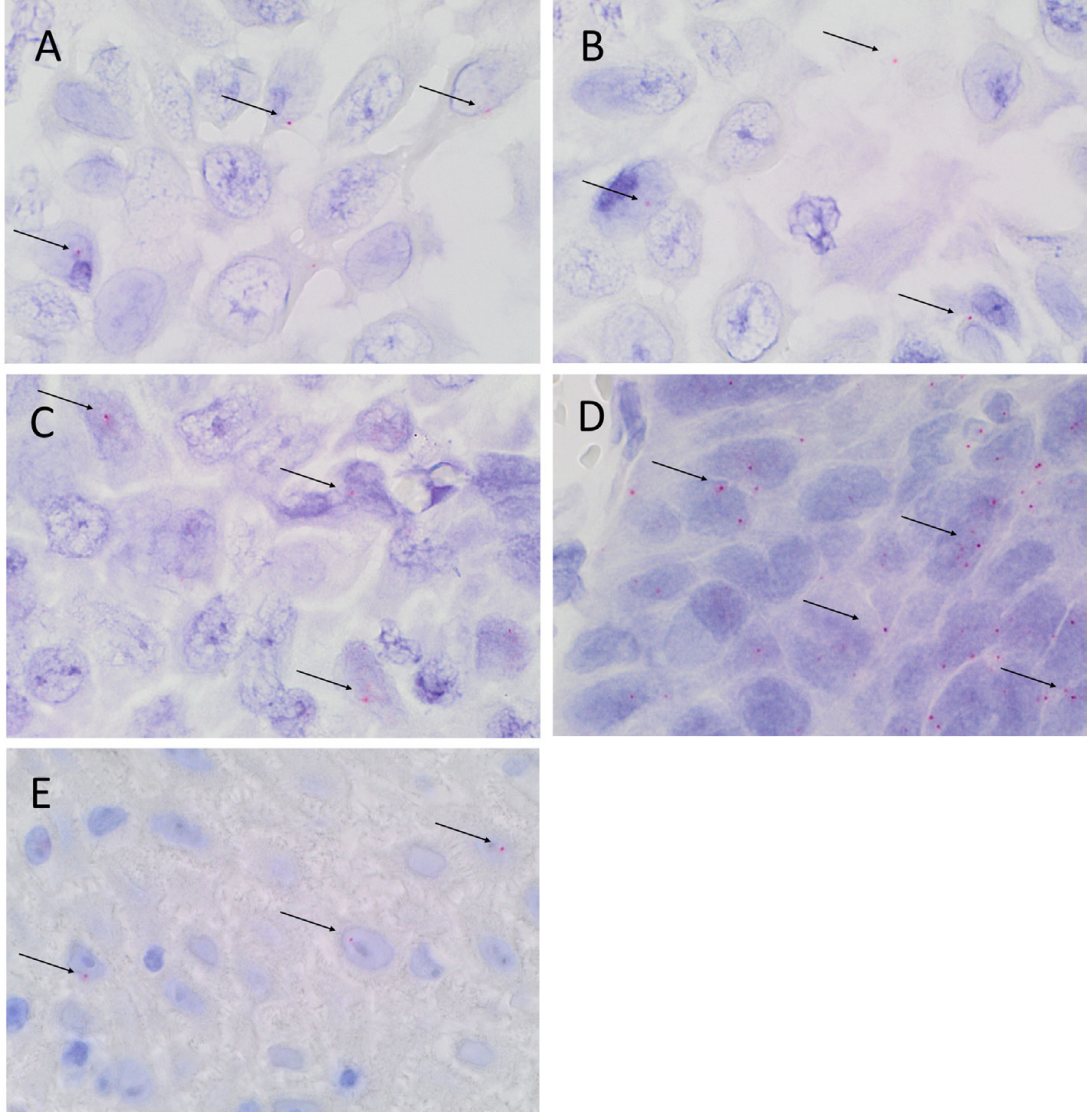
**Representative CISH-stained sections of MDBMSCC demonstrating mRNA expression of SOX2 [(A), pink, *arrows*], SALL4 [(B), pink, *arrows*], OCT4 [(C), pink, *arrows*], STAT3 [(D), pink, *arrows*], and NANOG [(E), pink, *arrows*]**. Original magnification: 1000×.

Colorimetric *in situ* hybridization confirmed the presence of mRNA for SOX2 (Figure 4A, pink, *arrows*), SALL4 (Figure 4B, pink, *arrows*), OCT4 (Figure 4C, pink, *arrows*), STAT3 (Figure 4D, pink, *arrows*), and NANOG (Figure 4E, pink, *arrows*) in the cells within all three MDBMSCC samples.

## Discussion

The recent literature supporting the concept of CSC in carcinogenesis has reported on the presence of these cells in multiple cancer types (12–14). The findings of this study further support previous reports on the presence of CSC in OCSCC (15, 36). The discovery of more than one subpopulation of putative CSC within MDBMSCC expressing ESC markers is novel. The detection of a EMA^+^/CD44^+^/SOX2^+^/OCT4^+^/SALL4^+^/pSTAT3^+^/NANOG^+^ subpopulation within the tumor nests aligns with recent literature reporting localization of CSC in OCSCC (15, 36), particularly at the “tumor front” (37). To the best of our knowledge, this is the first report demonstrating a EMA^−^/CD34^+^/CD44^−^/SOX2^+^/OCT4^+^/SALL4^+^/pSTAT3^+^/NANOG^+^ subpopulation on the endothelium of the microvessels, and a separate EMA^−^/CD44^−^/CD34^−^/SOX2^+^/OCT4^+^/SALL4^+^/pSTAT3^+^/NANOG^+^ subpopulation within the stroma of MDBMSCC. It is exciting to speculate that the abundant expression of these ESC markers, and therefore CSC, which are known to be associated with cell proliferation, invasion, and inhibition of cell death (19), is a reflection of the particularly aggressive nature of this cancer.

Interestingly, the ESC markers SOX2, OCT4, SALL4, pSTAT3, and NANOG, showed both nuclear and cytoplasmic expression. This novel finding in MDBMSCC is consistent with previous studies reporting on cytoplasmic expression of NANOG in cervical cancer (38) and SALL4 in breast cancer (39) cells, inferring cytoplasmic expression as a predictor of poor prognosis (39). The reasons for this are the topic of further investigation.

This novel demonstration of the co-expression of ESC markers within the endothelium of the microvessels and the cells within the stroma may be a result of an epithelial-to-mesenchymal transition (EMT) and vascular mimicry, which have been previously demonstrated within breast (40) and head and neck (41) cancers. It is exciting to speculate that the CSC in MDBMSCC undergo EMT and preferentially express OCT4, pSTAT3, SOX2, and NANOG and potentially lose the expression of EMA and CD44, as none of these cells outside of the tumor nests express EMA or CD44. A reason for this is the potential loss of the relatively more downstream markers EMA and CD44, as the cells adopt a more stem-like phenotype with the ability to undergo EMT (42). This would be consistent with a previous report demonstrating the increased expression of the EMT-associated genes, *twist* and *snail1*, in the stromal population of pharyngeal SCC (43). Alternatively, these three CSC subpopulations may be distinct from one another, each playing a vital role in carcinogenesis. However, this is beyond the scope of this study.

The expression of ESC markers by the endothelium of the microvessels demonstrated by IHC and CISH staining is fascinating and merits further investigation. Curiously, while all five ESC markers were demonstrated using IHC staining, NanoString analysis confirmed the presence of the mRNA transcripts CD44, NANOG, OCT4, SOX2, and STAT3 in all the MDBMSCC samples from all five patients, while SALL4 was detected in only four of the five samples. A possible reason for this is the heterogeneous nature of cancer tissues. The abundance of cells within MDBMSCC expressing stem cell markers demonstrated by us is consistent with similar reports on hypopharyngeal SCC (44). Our findings of OCT4 expression in the tumor nests is similar to that reported by Ge et al. (44) showing OCT4 staining within hypopharyngeal SCC. However, we have also demonstrated the presence of an independent OCT4^+^ subpopulation within the stroma, consistent with the findings of Huang et al. (16).

Our demonstration of the presence of three putative CSC subpopulations within MDBMSCC adds further insight into the biology of this disease, highlighting a potential vital role for CSC in tumor growth and development, locoregional and distant metastatic spread, and resistance to RT and ChT. A greater understanding of the CSC, by characterizing the subpopulations in well and poorly differentiated BMSCC lesions using these same markers, as well as further investigations into their microenvironment and their regulatory pathways, may reveal novel therapeutic targets for this aggressive cancer.

## Take Home Messages

(1) This study demonstrates the novel finding of three CSC subpopulations within MDBMSCC, with; (2) one CSC subpopulation within the tumor nests, expressing EMA, CD44, SOX2, SALL4, OCT4, pSTAT3, and NANOG; (3) a second CSC subpopulation is within the stroma between the tumor nests, expressing SOX2, SALL4, OCT4, pSTAT3, and NANOG; and (4) a third CSC subpopulation expressing CD34, SOX2, SALL4, OCT4, pSTAT3, and NANOG localized to the endothelium of the microvessels within the stroma.

## Ethics Approval

This study was approved by Central Regional Health and Disability Ethics Committee (ref. no: 12/CEN/74).

## Author Contributions

TI and STT formulated the study hypothesis. TI and STT designed the study. HHY, TF, HDB, and TI interpreted the IHC data. HHY, TF, HDB, and TI interpreted the CISH data. AMC processed the tissues for NanoString analysis and interpreted the data. HHY, TI, PFD, and STT drafted the manuscript. All authors commented on and approved the manuscript.

## Conflict of Interest Statement

The authors declare that the research was conducted in the absence of any commercial or financial relationships that could be construed as a potential conflict of interest. TI, PFD, and STT are inventors of the PCT patent application (No. PCT/NZ2015/050108) Cancer Diagnosis and Therapy.

## Supplementary Material

Figure S1 **DAB IHC-stained sections of human infantile hemangioma for NANOG [(A), brown] and SALL4 [(B), brown], myometrium for OCT4 [(C), brown], skin for SOX2 [(D), brown], tonsil for pSTAT3 [(E), brown] and CD44 [(F), brown], and the omission of the primary antibody in a moderately differentiated MDBMSCC sample provided an appropriate negative control [(G), brown]**. Cellular nuclei were counterstained with hematoxylin [**(A–F)**, blue]. Original magnification: 400×.

Figure S2 **Representative IF IHC-stained sections of MDBMSCC demonstrating nuclear expression of pSTAT3 [(A,C,M), red], EMA [(B), green], NANOG [(E), red], CD34 [(D,F,H), green], SOX2 [(G,I,K), red], SALL4 [(J), green], OCT4 [(L), green], and CD44 [(N), green]**. Separated images of the individual stains shown in **(A–N)**. Scale bars: 20 μm.
