# Supplementary figures and images for: Characterization of Cancer Stem Cells in Moderately Differentiated Buccal Mucosal Squamous Cell Carcinoma
Source: Front Surg. 2016 Aug 2;3:46. doi: 10.3389/fsurg.2016.00046 (PMC4970507; doi:10.3389/fsurg.2016.00046)

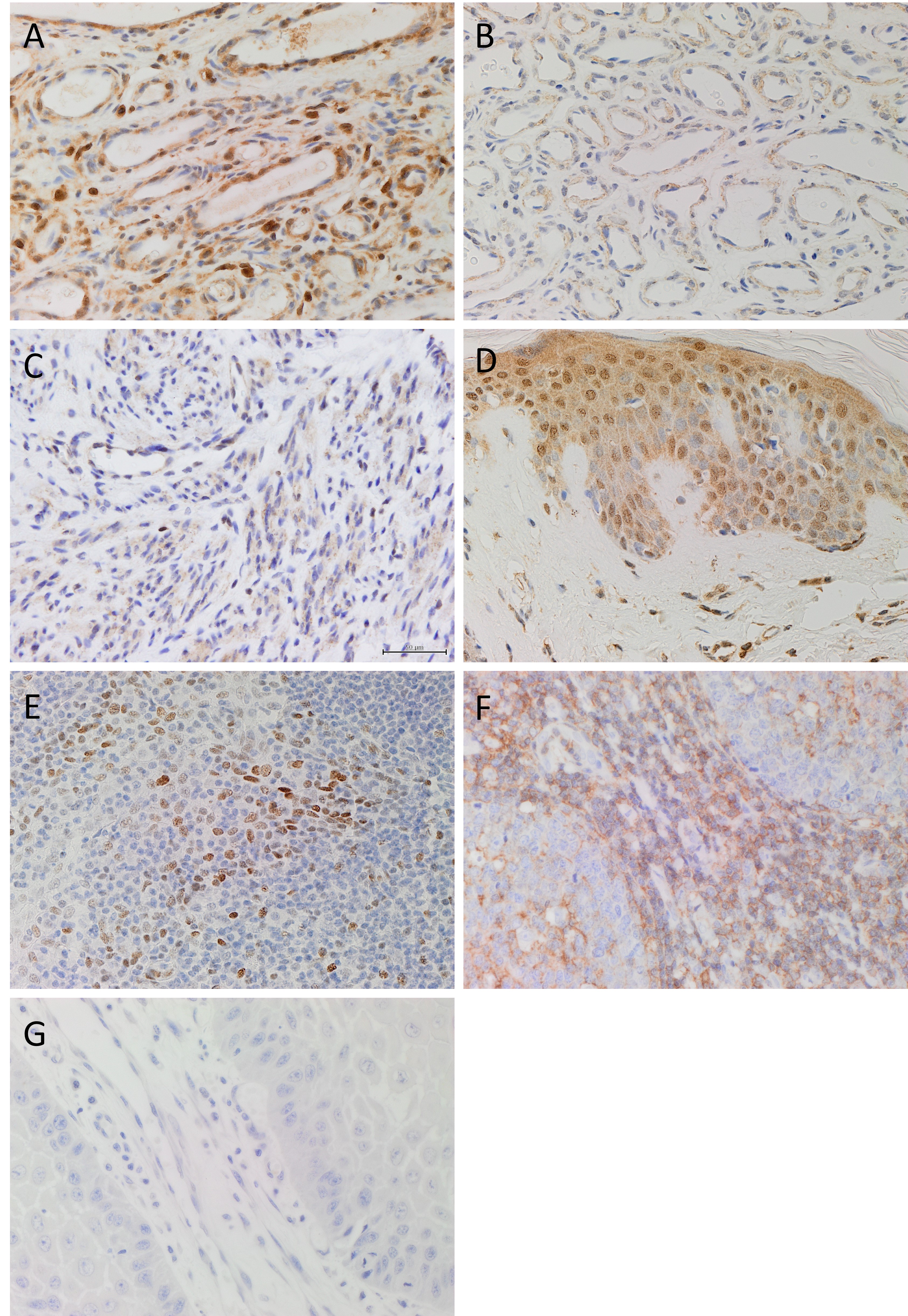

Supplement: Figure S1 — DAB IHC-stained sections of human infantile hemangioma for NANOG [(A), brown] and SALL4 [(B), brown], myometrium for OCT4 [(C), brown], skin for SOX2 [(D), brown], tonsil for pSTAT3 [(E), brown] and CD44 [(F), brown], and the omission of the primary antibody in a moderately differentiated MDBMSCC sample provided an appropriate negative control [(G), brown]. Cellular nuclei were counterstained with hematoxylin [(A–F), blue]. Original magnification: 400×. [file Image_1.JPEG]

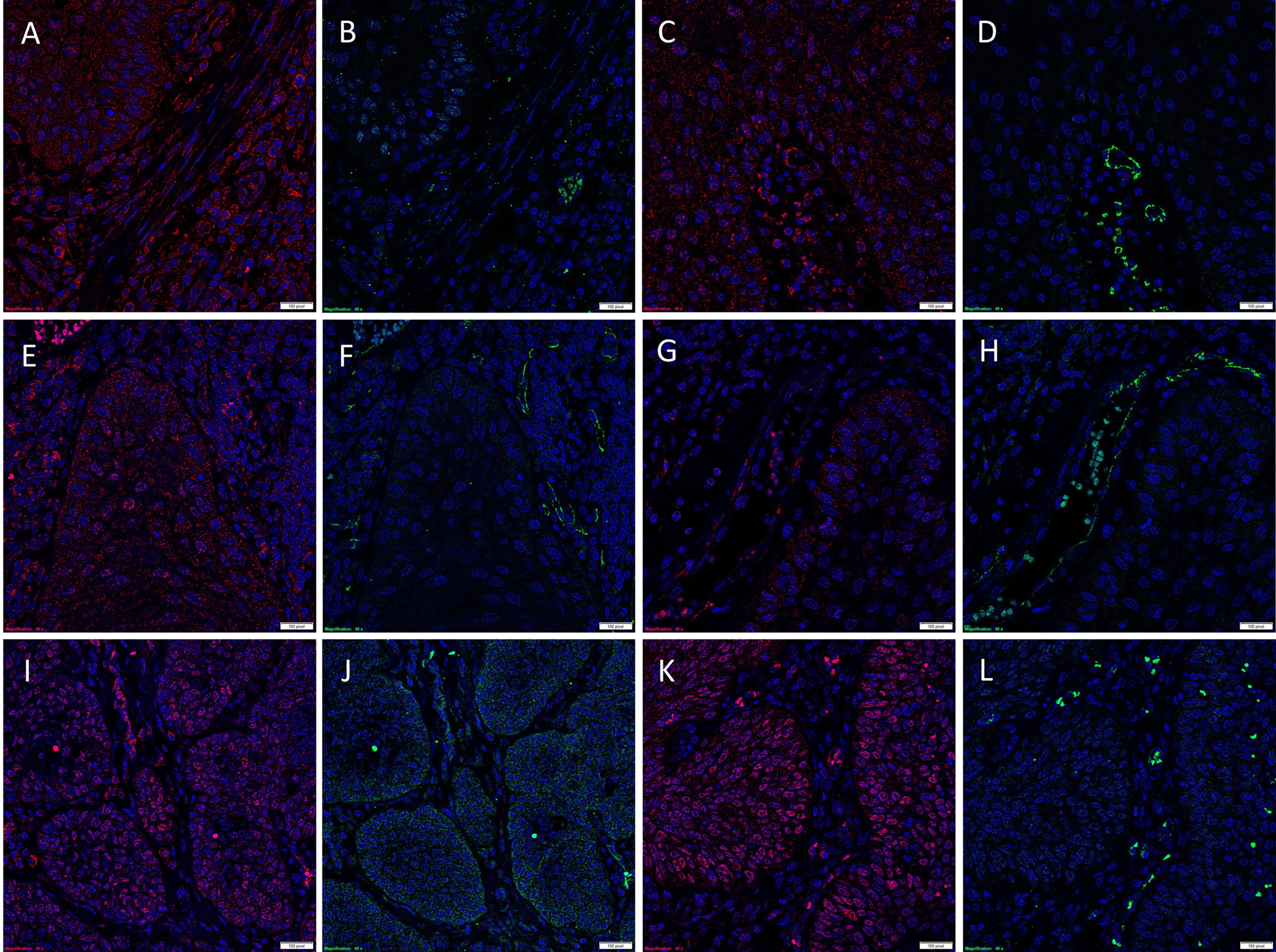

Supplement: Figure S2 — Representative IF IHC-stained sections of MDBMSCC demonstrating nuclear expression of pSTAT3 [(A,C,M), red], EMA [(B), green], NANOG [(E), red], CD34 [(D,F,H), green], SOX2 [(G,I,K), red], SALL4 [(J), green], OCT4 [(L), green], and CD44 [(N), green]. Separated images of the individual stains shown in (A–N). Scale bars: 20 μm. [file Image_2.JPG]
